# Air pollution diesel particles decrease synaptosomal post‐synaptic but not pre‐synaptic proteins in mouse cortex

**DOI:** 10.1002/alz.095780

**Published:** 2025-01-09

**Authors:** Jose A Godoy‐Lugo, Max A Thorwald, Constantinos Sioutas, William J Mack, Caleb E Finch

**Affiliations:** ^1^ USC Leonard Davis School of Gerontology, Los Angeles, CA USA; ^2^ USC Viterbi School of Engineering, Los Angeles, CA USA; ^3^ USC Zilkha Neurogenetic Institute, Los Angeles, CA USA; ^4^ USC Dornsife College of Letters, Arts, and Sciences, Los Angeles, CA USA

## Abstract

**Background:**

Chronic air pollution exposure increases accelerates cognitive aging and AD risk. Synapse loss in AD correlates with decreased cognitive ability. In rodents, inhaled air pollutants decreased glutamatergic synapses, decreasing excitatory postsynaptic currents (EPSCs), while increasing total levels of AMPA and NMDA receptor protein. To further resolve air pollution effects in these synaptic components, we examined synaptosomal proteins from mice exposed to diesel exhaust particles (DEP).

**Method:**

C57BL/6 mice were exposed to re‐aerosolized DEP (n = 12, 100 µg/m^3^; NIST 2975) or filtered air (FA, n = 12) for 5 h/day for 8 weeks. Synaptosomes were isolated by centrifugation from cerebral cortex and proteins analyzed by immunoblots.

**Result:**

DEP exposure decreased synaptosomal PSD95 by ‐30% and Homer1, and SAP97 by ‐20%. Also decreasing NMDA receptor subunits GluN1 and GluN2A by ‐20%. In contrast, DEP did not alter pre‐synaptic vesicle proteins SNAP‐25, SYN1, SYT1, and SYP.

**Conclusion:**

Mechanisms behind early synapse disfunction after exposure to air pollution may relate to greater decreases in post‐synaptic than the pre‐synaptic proteins we assayed at the synapse. Future studies will evaluate the hippocampus, which has shown selective impact in CA1 neurons EPSCs, and the role of air pollution‐induced oxidative damage at synapse cites.

